# How to Feed Children

**Published:** 1896-08

**Authors:** 


HOW TO FEED CHILDREN. A Manual for Mothers, Nurses and Physicians. By LOUISE E. HOGAN. Price, $1.00. Philadelphia: J. B. Lippincott Company. 1896.

This hand-book belongs in that excellent series, Practical lessons in nursing. It includes an instructive chapter on infant feeding, in which the mother is wisely referred to her physician for the proper formula in the individual case, while she is shown the method of sterilising, the selection and care of the nursing bottle, and the like. The various classes of foods are taken up in detail, with their nutritive value, the best methods of preparing and the age at which they may be given. Dietaries and menus, summer diet, and diet in illness, are each given a chapter. Every physician who reads the book will feel that in recommending it to mothers he suggests a safe guide, which, if followed, will lessen the frequency of gastro-intestinal disorders among the young.

